# Do Intracerebral Cytokine Responses Explain the Harmful Effects of Dexamethasone in Human Immunodeficiency Virus–associated Cryptococcal Meningitis?

**DOI:** 10.1093/cid/ciy725

**Published:** 2018-08-30

**Authors:** Justin Beardsley, Nhat L T Hoang, Freddie M Kibengo, Nguyen L N Tung, Tran Q Binh, Le Q Hung, Wirongrong Chierakul, Guy E Thwaites, Nguyen V V Chau, Thuong T T Nguyen, Ronald B Geskus, Jeremy N Day

**Affiliations:** 1 Oxford University Clinical Research Unit, Ho Chi Minh City, Vietnam; 2 Centre for Tropical Medicine and Global Health, Nuffield Department of Medicine, Oxford, United Kingdom; 3 Marie Bashir Institute, University of Sydney, New South Wales, Australia; 4 MRC Masaka and MRC/UVRI & LSHTM Uganda Research Unit, Entebbe; 5 Hospital for Tropical Diseases, Ho Chi Minh City, Vietnam; 6 Department of Tropical Medicine, Cho Ray Hospital, Ho Chi Minh City, Vietnam; 7 Mahidol Oxford Tropical Medicine Research Unit, Mahidol University, Bangkok, Thailand

**Keywords:** cryptococcal, corticosteroids, cytokines, LTA4H, dexamethasone

## Abstract

**Background:**

The CryptoDex trial showed that dexamethasone caused poorer clinical outcomes and slowed fungal clearance in human immunodeficiency virus–associated cryptococcal meningitis. We analyzed cerebrospinal fluid (CSF) cytokine concentrations from participants over the first week of treatment to investigate mechanisms of harm and test 2 hypotheses: (1) dexamethasone reduced proinflammatory cytokine concentrations, leading to poorer outcomes and (2) leukotriene A4 hydrolase (LTA4H) genotype influenced the clinical impact of dexamethasone, as observed in tuberculous meningitis.

**Methods:**

We included participants from Vietnam, Thailand, and Uganda. Using the Luminex system, we measured CSF concentrations of the following: interferon γ, tumor necrosis factor (TNF) α, granulocyte-macrophage colony-stimulating factor, monocyte chemoattractant 1, macrophage inflammatory protein 1α, and interleukin 6, 12p70, 8, 4, 10, and 17. We determined the LTA4H genotype based on the promoter region single-nucleotide polymorphism rs17525495. We assessed the impact of dexamethasone on cytokine concentration dynamics and the association between cytokine concentration dynamics and fungal clearance with mixed effect models. We measured the influence of LTA4H genotype on outcomes with Cox regression models.

**Results:**

Dexamethasone increased the rate TNF-α concentration’s decline in (−0.13 log2pg/mL/d (95% confidence interval, −.22 to −.06 log2pg/mL/d; P = .03), which was associated with slower fungal clearance (correlation, −0.62; 95% confidence interval, −.83 to −.26). LTA4H genotype had no statistically significant impact on outcome or response to dexamethasone therapy. Better clinical outcomes were associated with higher baseline concentrations of interferon γ.

**Conclusions:**

Dexamethasone may slow fungal clearance and worsen outcomes by increasing TNF-α concentration’s rate of decline.

## Teaser

We investigated clinical and microbiological impacts of longitudinal cerebrospinal fluid cytokine concentrations in human immunodeficiency virus–associated cryptococcal meningitis, and interactions with dexamethasone therapy or leukotriene A4 hydrolase genotype. We found that faster declines in tumor necrosis factor α were associated with reduced fungal clearance.

Human immunodeficiency virus (HIV)–associated cryptococcal meningitis (CM) affects approximately 230000 people annually, mostly in low- and low-middle income countries [1]. Even with optimal antifungal treatment, outcomes are poor, with 10-week mortality rates of about 30% [1–4]. Because both pathogen and host immune responses contribute to disease phenotype [5], we wanted to investigate whether adjunctive management of inflammation could reduce these unacceptable mortality rates. To this end, we conducted a randomized placebo-controlled trial of dexamethasone in CM [6].

The rationale for the trial, described in the protocol [7], was that CM is often associated with inflammatory complications which can include increased intracranial pressure, cerebral edema, and vasculitis [8–11]. Corticosteroids are potent immune modulators, reducing production of proinflammatory cytokines, including interferon (IFN) γ [12, 13], and are indicated to treat each of these complications in other clinical conditions. We hypothesized that dexamethasone would reduce inflammatory complications whilst optimal antifungal therapy cleared the infection. Our hypothesis was supported by retrospective data suggesting that corticosteroids reduce the risk of blindness in HIV-uninfected patients with CM [14], and by animal models of CM showing that dexamethasone improves survival without impairing the sterilizing power of antifungals [15, 16]. Furthermore, human clinical trials have shown dexamethasone can improve clinical outcomes in other forms of meningitis, including adults with tuberculous meningitis (TBM) [17] and acute bacterial meningitis [18, 19].

Not all data supported our hypothesis. Studies prior to our trial demonstrated that both higher endogenous baseline IFN-γ cerebrospinal fluid (CSF) concentrations and administration of IFN-γ were associated with faster fungal clearance in HIV-associated CM [20, 21]. After our trial, data emerged showing an association between baseline proinflammatory cytokine clusters and reduced early mortality rates [22]. However, very few data exist describing how cytokine concentrations change over time and how this relates to clinical outcomes. Our trial was an opportunity to examine these relationships and to describe how dexamethasone affects cytokine profiles in patients established on effective antifungal therapy.

The CryptoDex trial showed dexamethasone was harmful in HIV-associated CM [6]. It stopped early, after the third safety analysis, because dexamethasone caused more disability and adverse events and slowed CSF sterilization. In the current study, to understand these harmful effects, we compared CSF cytokine dynamics over the first week of treatment between patients receiving dexamethasone or placebo. Our main hypotheses were that dexamethasone reduced proinflammatory cytokines, leading to the poorer clinical outcomes and slower fungal clearance observed in the trial.

Leukotriene A4 hydrolase (LTA4H) genotype has been shown to affect the inflammatory phenotype and clinical response to dexamethasone therapy in Vietnamese adults with TBM: CC homozygotes have a hypoinflammatory response, CT heterozygotes have a moderate inflammatory response, whereas TT homozygotes have a hyperinflammatory response. Given that patients with TBM with the TT genotype responded especially well to dexamethasone [23–25], we further hypothesized that patients with CM who have this genotype would benefit from dexamethasone.

## METHODS

### Study Design

We used CSF and blood samples from the CryptoDex trial [6], in which participants were randomized 1:1 to dexamethasone or placebo. Dexamethasone was administered intravenously for 2 weeks (0.3 mg/kg/d in week 1 and 0.2 mg/kg/d in week 2), then orally to complete 6 weeks in total (0.1 mg/kg/d in week 3, and 3, 2, and 1 mg/d, respectively in weeks 4, 5, and 6). The study received ethical approval from the institutional review boards at each site and the Oxford University Tropical Research Ethics Committee. All patients gave written informed consent, which included use of clinical samples for further research, and an opt-in clause for human genetic testing.

### Participants

We recruited participants with HIV infection, a syndrome consistent with CM, and ≥1 of the following: positive CSF India ink stain, positive CSF or blood culture for *Cryptococcus*, or positive CSF cryptococcal antigen (IMMY Cryptococcal Antigen Lateral Flow Assay; Immuno-Mycologics). Specific exclusions are detailed in the trial protocol [7].

We had ethical approval to measure the CSF cytokine concentrations of CryptoDex participants recruited in Uganda and Vietnam (n = 308). Baseline analyses included all participants with CSF samples available from a baseline lumbar puncture. Longitudinal analyses included all those with CSF samples available from study entry, study day 1–2, or study day 4–7. We performed LTA4H genotyping on all consenting patients from Vietnam, Uganda, and Thailand.

### Procedures

Participants received inpatient care for the first 2 weeks, and longer if necessary. Lumbar punctures were performed on days 1, 3, 7, and 14, and more frequently if indicated. Ambulant participants were discharged after 2 weeks, with follow-up at weeks 3, 6, and 10 and month 6.

CSF samples were stored at −80^o^C. We measured the concentrations of IFN-γ, tumor necrosis factor (TNF) α, granulocyte-macrophage colony-stimulating factor, interleukin 6, interleukin 12p70, interleukin 8, monocyte chemoattractant protein 1, macrophage inflammatory protein 1α, interleukin 4 and 10 (IL-4 and IL-10), and interleukin 17 using R&D multiplex human cytokine kits (R&D Systems), on the Luminex platform (Luminex). We determined LTA4H genotype using a validated TaqMan assay of the LTA4H promoter region single-nucleotide polymorphism (rs17525495) [26].

### Outcomes

The main outcomes of interest were the rate of change in CSF of IFN-γ, TNF-α, IL-4, and IL-10 concentrations and IFN-γ/IL-4 and TNF-α/IL-10 ratios over the first 7 days of treatment. Other cytokines were considered exploratory variables. Clinical end points included survival and disability by 10 weeks and 6 months. We categorized disability outcomes as described elsewhere [17] (Supplementary Table 1). Our mycological outcome was the rate of fungal clearance over the first 2 weeks (early fungicidal activity [EFA]).

### Statistical Methods

Analyses were predetermined in a statistical analysis plan, unless otherwise stated, and were performed using R software, version 3.2.1 [27]. To demonstrate that this study sample was representative of the overall CryptoDex sample, we summarized the baseline clinical characteristics of those included and those not included. We compared baseline cytokine concentrations by clinical outcomes (good, intermediate disability, severe disability, or death) at 10 weeks and 6 months using the Wilcoxon rank sum test.

All cytokine measurements on days 0– 7 were included in the longitudinal analyses. Cytokine results below the limit of detection were left censored. We compared the change in CSF cytokine concentrations over the first 7 days by treatment arm using a linear mixed-effect model (uncorrected for truncation by early death). We included an interaction term between the treatment arm and time since enrollment. We allowed for random patient-specific intercepts and slopes. To increase the power of the models, we included cytokine concentrations from all CSF samples collected before randomization. We used these values to model cytokine concentration by duration since disease onset, using restricted cubic splines with 5 degrees of freedom. Modeling was done with the R package lmec, version 1.0 [28].

For IFN-γ, the majority (55%) of IFN-γ concentrations were already under the detection limit (which averages 1.27 pg/mL [29]) at baseline, so we dichotomized this variable at 30 pg/mL, based on a previous study where the lower quartile of baseline IFN-γ CSF concentrations was >30 pg/mL for patients surviving to 10 weeks [30]. We analyzed this variable in a generalized mixed effect model (using R package lme4, version 1.1–17 [31]), based on the regression formula used in the linear mixed model used for other cytokines. However, changing from a continuous to a binary variable caused a loss of power, and we were thus only able to use 4 degrees of freedom for the restricted cubic splines to model the nonlinear pattern of the odds of retaining IFN-γ concentrations ≥30 pg/mL over time. For this, and all other analyses, we removed cytokine results outside the 0.005–0.995 quantile range.

We assessed the impact of changes in CSF cytokine concentrations over the first 7 days on mortality rates at 10 weeks and 6 months, using a logistic regression model. The main covariates were treatment arm and the individual estimated rate of change in log_2_ cytokine concentrations, as derived from the linear mixed model above. The analyses were adjusted for the potential confounders baseline CSF fungal count and Glasgow Coma Scale score [3]. We estimated the correlation between the random effect of the rate of change in CSF cytokine concentrations and EFA based on a bivariate linear mixed model of all documented cytokine concentrations and fungal counts from the 7 days after randomization.

We compared baseline CSF white blood cell counts, fungal counts, and cytokine concentrations between patients with the CC, CT, and TT LTA4H genotypes, using the Wilcoxon rank sum test. We assessed the difference in estimated change in log_2_ cytokine concentration between the 3 LTA4H genotypes with a linear mixed-effect model, similar to the one described above. In this particular model, time and genotype were the main covariates. We included a 3-way interaction between time, genotype, and treatment arm.

We showed the impact of LTA4H on time to death with Kaplan-Meier curves by treatment arm, stratified by genotype. We assessed the effect of dexamethasone on time to death, and how this varied by genotype, with a Cox regression adjusted for country of enrollment. We added a time-varying coefficient (0–21 and 22–70 for 10 weeks and 0–21, 22–43, and 44–180 for 6 months) based on the observed nonproportional hazards of mortality seen in CryptoDex [6]. We used multiple imputation, with the R package mice [32], to deal with missing covariate values.

We used the Hochberg procedure (R function multtest [33]) to correct for multiple testing in the analyses of dexamethasone’s impact on cytokine concentrations. For the analyses of the impact of baseline features on 10-week mortality rates, we adjusted the *P* values for all regression coefficients based on multivariate *t* distribution, using the R package multcomp [34]. Both methods result in conservative *P* values; therefore, we only corrected results relating to the main hypotheses.

## RESULTS

**Figure 1.**
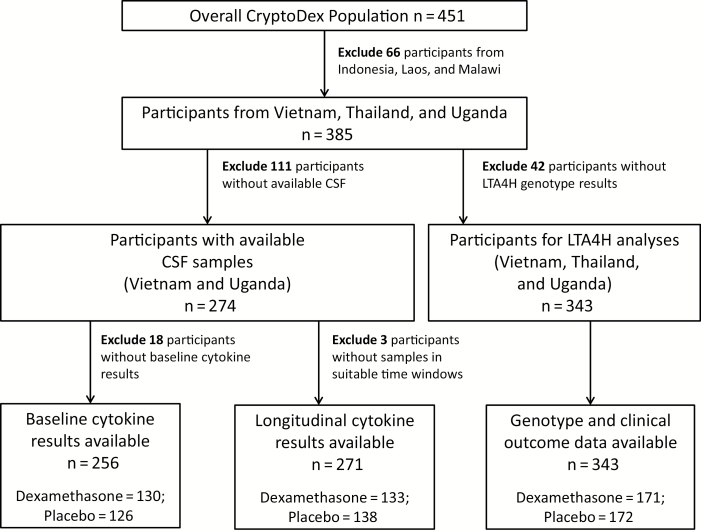
Study flowchart for the 3 main analysis groups in the study of dynamic immune responses in human immunodeficiency virus–associated cryptococcal meningitis. Abbreviations: CSF, cerebrospinal fluid; LTA4H, leukotriene A4 hydrolase.

Between February 2013 and August 2014, we recruited 451 participants (385 from Vietnam, Uganda, and Thailand). Of these, 343 gave consent for genetic testing and had samples available. Of 274 participants from Vietnam and Uganda who had stored CSF samples, 256 had baseline samples, and 271 had eligible longitudinal samples (Figure 1).

### Baseline Features

The population sample for this study was similar to the residual CryptoDex population (Supplementary Table 2). Table 1 details baseline CSF cytokine concentrations by disability and death at 10 weeks (for 6 months, see Supplementary Table 3). IFN-γ concentrations >30 pg/mL were more common in patients with good (34%) or intermediate (38%) outcomes than in those with severe disability (13%) or death (22%) (*P* = .02) by 10 weeks, and the trend was still evident at 6 months (*P* = .051). The concentration of monocyte chemoattractant 1 was significantly associated with clinical outcome at 10 weeks but not at 6 months, whereas interleukin 17 was associated with clinical outcome at 6 months but not at 10 weeks. Furthermore, neither displayed a clear dose response trend over both time points. Auxiliary logistic regression of 10-week mortality rates by baseline cytokine concentration (Supplementary Table 4) also showed that participants with a baseline IFN-γ concentration >30 pg/mL had a reduced risk of death (odds ratio, 0.38; 95% confidence interval [CI], 15–.91; unadjusted *P* = .03). This result lost statistical significance after adjustment for multiple testing (adjusted *P* = .50).

**Table 1.** Baseline Cytokine Concentrations by Clinical Outcome at 10 Weeks

Cytokine Concentration	Clinical Outcome at 10 wk				*P* Value^a^
	Good (n = 32)	Intermediate n = 69)	Severe Disability (n = 38)	Death (n = 113)	
IFN-γ >30 pg/mL, No. of Patients (%)	11 (34)	26 (38)	5 (13)	24/110 (22)	.02
Cytokine concentration, median (IQR), pg/mL					
TNF-α	43.41 (20.53–89.26)	59.71 (28.05–178.53)	43.41 (17.75–109.14)	51.98 (17.75–138.14)	.54
MCP-1	1016.93 (630.35–2521.38)	1217.75 (719.08–2048)	2134.97 (744.43–4153.18)	1807.78 (867.07–4096)	.03
MIP-1a	613.11 (388.02–873.1)	694.58 (418.77–1136.2)	781.44 (467.88–1176.27)	680.29 (433.53–1060.11)	.80
GM-CSF	3.41 (1.78–9)	4.56 (2.45–13.09)	3.2 (1.47–8.28)	3.27 (1.11–11.96)	.44
IL-6	99.04 (42.52–922.88)	354.59 (71.01–1067.48)	99.73 (27.1–588.13)	126.24 (33.13–430.54)	.11
IL-8	1009.9 (364.56–3565.78)	1398.83 (592.22–3929.15)	1016.93 (340.14–3565.78)	1192.69 (436.55–2797.65)	.72
IL-12	6.5 (2.93–9.85)	7.57 (2.93–11.24)	6.96 (2.93–11.88)	7.16 (2.93–10.56)	.80
IL-4	23.43 (16.22–31.34)	25.46 (16.22–33.13)	23.26 (16.45–27.86)	24.76 (16.8–31.34)	.79
IL-10	7.67 (2.41–13.09)	13.74 (4.66–39.4)	11.88 (3.39–35.26)	9.38 (2.95–23.43)	.13
IL-17	6.92 (4–15.03)	7.21 (3.97–27.67)	9.85 (4.14–15.35)	6.41 (3.16–9.19)	.07

Abbreviations: GM-CSF, granulocyte-macrophage colony-stimulating factor; IFN, interferon; IL-4, IL-6, IL-8, IL-10, IL-12, and IL-17, interleukin 4, 6, 8, 10, 12, and 17; IQR, interquartile range; MCP, monocyte chemoattractant; MIP, macrophage inflammatory protein; TNF, tumor necrosis factor.

^a^ *P* values based on the Wilcoxon rank sum test for continuous data and the χ^2^ test for categorical data.

### Impact of Dexamethasone on Cytokine Concentrations

**Figure 2.**
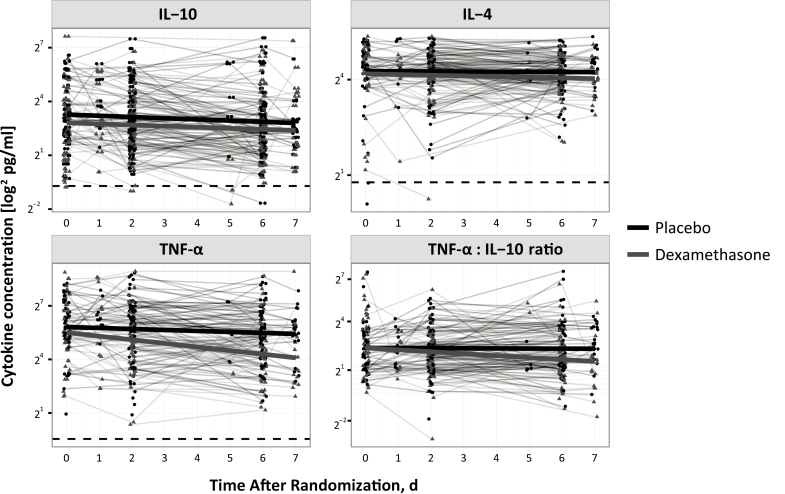
Concentrations of interleukin 10 (IL-10), interleukin 4 (IL-4), tumor necrosis factor (TNF) α, and TNF-α/IL-10 ratio over the first 7 days after randomization. Data from patients receiving placebo are shown in black, and data from those receiving dexamethasone in grey. Bold lines in black and grey are the linear regressions from the univariate model; dashed lines, the lower limit of detection for each cytokine.

Patients receiving dexamethasone had faster rates of decline in TNF-α concentrations over the first 7 days of treatment (difference in slope, −0.13 log_2_ pg/mL/d; 95% CI, −.22 to −.06; adjusted *P* = .03) (Figure 2 and Table 2). Furthermore, dexamethasone was associated with more rapid declines in the CSF TNF-α/IL-10 ratio (−0.14/d; 95% CI, −.21 to −.06; adjusted *P* < .001).

**Table 2.** Results of Univariate Mixed Model of Longitudinal Cytokine Concentrations by Treatment Arm

Cytokine	Impact of Dexamethasone on Cytokine Concentration Slope (95% CI)	Unadjusted *P* Value	Adjusted *P* Value^a^
IFN-γ, log_2_ OR/d^b^	−0.02 (−1.26 to 1.22)	.97	.97
Cytokine, log_2_ pg/mL/d			
TNF-α	−0.13 (−.22 to −.06)	.007	.03
IL-4	−0.01 (−.06 to .04)	.64	.97
IL-10	0.008 (−.09 to.10)	.88	.97
Log_2_ TNF-α/IL-10 ratio/d	−0.14 (−.21 to −.06)	<.001	<.001

Abbreviations: CI, confidence interval; IFN, interferon; IL-4, interleukin 4, IL-10, interleukin 10; OR, odds ratio; TNF, tumor necrosis factor.

^a^ *P* values adjusted using the Hochberg method.

^b^OR of IFN-γ concentration being >30 pg/mL.

### Impact of Changes in Cytokine Concentration on Clinical and Microbiological Outcomes

We used a logistic regression model to assess whether the rate of CSF cytokine concentration decline had an impact on mortality rates at 10 weeks or 6 months. With adjustment for dexamethasone therapy, baseline Glasgow Coma Scale score, baseline fungal count, and multiple testing, we found no statistically significant effect of IFN-γ, TNF-α, IL-4, or IL-10 clearance on mortality rates (see Table 3). Adding an interaction term for dexamethasone made no difference. However, we did find a strong negative correlation between rate of decline in IL-10 and EFA, and a moderate negative correlation between both TNF-α and IL-4 and EFA (ie, faster rates of decline were associated with slower rates of fungal clearance; Supplementary Table 5).

**Table 3.** Results of Logistic Regression on Cytokine Slope for 10-Week and 6-Month Mortality Rates

Cytokine Slope	10-wk Mortality Rate (n = 253)^a^			6-mo Mortality Rate (n = 252)^a^		
	OR (95% CI)	Unadjusted *P* Value	Adjusted *P* Value^b^	OR (95% CI)	Unadjusted *P* Value	Adjusted *P* Value^b^
IFN-γ, log_2_ OR/d^c^	1.31 (1.01–1.71)	.04	.20	1.22 (.96–1.56)	.10	.52
Cytokine concentration, log_2_ pg/mL/d						
TNF-α	0.92 (.05–15.74)	.96	.96	0.55 (.03–9.58)	.68	.68
IL-4	0.24 (.005–11.12)	.47	.96	7.01 (.18–261.18)	.29	.68
IL-10	3.90 (.16–94.69)	.40	.96	3.07 (.13–71.24)	.48	.68
TNF-α/IL-10 log2ratio/d	0.68 (0.12–3.76)	.65	.96	0.45 (0.08–2.50)	.36	.68

Abbreviations: CI, confidence interval; IFN, interferon; IL-4, interleukin 4; IL-10, interleukin 10; OR, odds ratio; TNF, tumor necrosis factor.

^a^Three patients were removed from the 10-week and 4 from the 6-month analysis, owing to inadequate data.

^b^ *P* values adjusted using the Hochberg method.

^c^OR of IFN-γ concentration being >30 pg/mL.

### Impact of LTA4H Genotype

Twenty patients (6%) had the TT genotype, 122 (36%) had TC, and 201 (59%) had CC. The TT genotype was significantly more common among participants in Asia than among those in Uganda (18 of 171 [10.5%] vs 2 of 172 [1.2%]; *P* < .001).

We compared baseline CSF features between genotypes and found that fungal counts were lower in patients with the TT genotype (3.44 [95% CI, 2.6–4.87] log_10_ colony-forming units [CFUs]/mL) than in those with the CT (4.92 [3.05–5.8] log_10_ CFUs/mL) or CC (4.04 [1.9 –5.43] log_10_ CFUs/mL) genotype (*P* = .004) (Supplementary Table 6). The pattern was similar when stratified by continent (Supplementary Table 7). However, we did not see a significant effect of genotype on CSF white blood cell count. In terms of the genotype’s impact on cytokines, although baseline concentrations of IFN-γ and TNF-α seemed to be higher in the TT group, this difference was not statistically significant (Supplementary Table 6). We also compared changes in cytokine concentration over time and cytokine response to dexamethasone between the genotype groups, and we found no significant effect (Table 4).

**Table 4.** Results of Univariate Mixed Model of Longitudinal Cytokine Concentration, Genotype, and Treatment Arm

Cytokine^a^	TT vs CT, Difference in Slope (95% CI), Log_2_ pg/mL	*P* Value	TT vs CC Difference in Slope (95% CI), Log_2_ pg/mL	*P* Value	Impact of Dexamethasone on TT vs CT Cytokine Concentration Slope, Difference (95% CI), Log_2_ pg/mL	*P* Value	Impact of Dexamethasone on TT vs CC Cytokine Concentration Slope, Difference (95% CI), Log_2_ pg/mL	*P* Value
TNF-α	−0.01 (−.41 to .38)	.95	−0.09 (−.47 to .28)	.63	0.37 (−.09 to .85)	.12	0.33 (−.12 to .79)	.15
MCP-1	0.12 (−.2 to .45)	.46	0.17 (−.14 to .48)	.28	−0.07 (−.46 to .32)	.73	−0.09 (−.47 to .3)	.66
MIP-1a	0.04 (−.19 to .27)	.73	−0.0007 (−.21 to .21)	.99	0.02 (−.25 to .3)	.86	−0.01 (−.28 to .26)	.92
GM-CSF	−0.16 (−.59 to .27)	.48	−0.26 (−.67 to .15)	.21	1.37 (−.38 to 3.12)	.13	1.5 (−.27 to 3.22)	.10
IL-6	−0.20 (−.98 to .58)	.61	−0.28 (−1.03 to .47)	.46	0.38 (−.45 to 1.22)	.37	0.33 (−.47 to 1.14)	.41
IL-8	−0.17 (−.61 to .25)	.43	−0.18 (−.59 to .23)	.39	0.20 (−.31 to .71)	.44	0.17 (−.32 to .66)	.50
IL-12	−0.13 (−.35 to .09)	.25	−0.11 (−.32 to .09)	.29	0.45 (−.48 to 1.38)	.34	0.50 (−.42 to 1.43)	.29
IL-4	0.08 (−.29 to .13)	.46	−0.05 (−.25 to .15)	.63	−0.01 (−.26 to .23)	.91	0.04 (−.2 to .27)	.76
IL-10	−0.05 (−.45 to .35)	.81	−0.19 (−.57 to .19)	.32	0.21 (−.28 to .72)	.39	0.27 (−.22 to .75)	.28
IL-17	0.08 (−.28 to .44)	.67	−0.003 (−.35 to .34)	.99	−0.19 (−.59 to .21)	.35	−0.24 (−.62 to .15)	.22

Abbreviations: CI, confidence interval; GM-CSF, granulocyte-macrophage colony-stimulating factor; IL-4, IL-6, IL-8, IL-10, IL-12, and IL-17, interleukin 4, 6, 8, 10, 12, and 17; MCP, monocyte chemoattractant; MIP, macrophage inflammatory protein; TNF, tumor necrosis factor.

^a^Interferon γ was excluded because dichotomization led to a lack of power to detect differences between genotypes.

LTA4H genotype was not significantly associated with mortality rate, taking all patients into consideration. The logistic regression of mortality rates by genotype gave an odds ratio for death at 10 weeks of 0.58 (95% CI, .08–3.93) for CT vs TT, and 0.67 (.10–4.28) for CC vs TT (Supplementary Table 4).

**Figure 3.**
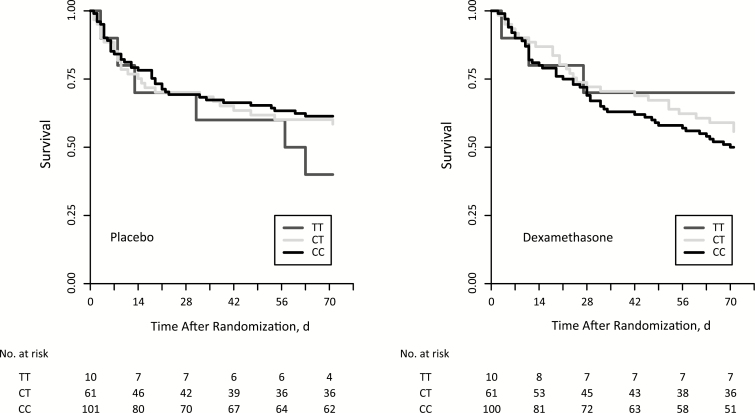
Kaplan-Meier curves of survival up to 10 weeks for CC (black), CT (light grey), and TT (dark grey) genotypes by placebo and dexamethasone treatment arms.

We looked for a genotype-specific effect of dexamethasone on mortality, but though the Kaplan-Meier charts (Figure 3 and Supplementary Figure 1) suggest that dexamethasone may reduce the mortality rate for patients with the TT genotype, we lacked power to demonstrate this statistically (hazard ratio [HR] for mortality from day 21 to day 70, 0.41; 95% CI, .02–7.45). In contrast, dexamethasone’s deleterious effect on mortality between days 21 and 70 was statistically significant for patients with CC (HR, 3.24; 95% CI, 1.31–8.04) and CT (3.41; 1.07–10.90) genotypes (see Table 5). We observed the same pattern in the 6-month analysis, where there was clear evidence for a delayed increase in the mortality HR for CT and CC groups, but not for TT (Supplementary Table 8).

**Table 5.** HRs from Cox Regression on 10-Week Mortality Rate Related to Dexamethasone Therapy by LTA4H Genotype, with Time-Dependent Variable to Account for Nonproportional Hazards

Genotype	Time-Dependent HR for Mortality Rate Related to Dexamethasone Therapy (95% CI)^a^	
	d 0–21	d 21–70
All	0.73 (.48–1.12)	2.87 (1.45–5.66)
CC	0.83 (.48–1.42)	3.24 (1.31–8.04)
CT	0.59 (.28–1.22)	3.41 (1.07–10.90)
TT	0.58 (.1–3.59)	0.41 (.02–7.45)

Abbreviations: CI, confidence interval; HR, hazard ratio; LTA4H, leukotriene A4 hydrolase.

^a^Analysis corrected for participant’s country of origin.

## DISCUSSION

We described the effect of dexamethasone and LTA4H genotype on longitudinal CSF cytokine concentrations in HIV-associated CM, aiming to determine whether variations in these factors could explain the harmful effects of dexamethasone. We showed that faster rates of decline in TNF-α concentration were correlated with reduced EFA. Although we lacked the power to demonstrate any mortality effect in our participants, the effect of EFA on mortality rate is already well established [35]. We showed that dexamethasone caused more rapid declines in TNF-α concentration, and this provides a potential explanation for dexamethasone’s harmful effects on fungal clearance. Our observations are consistent with data from mice, in which TNF-α deficiency at the time of infection causes suboptimal activation of dendritic cells [36] and is associated with failure to clear infection [37]. Depletion of TNF-α is known to predispose humans to other invasive fungal infections [38]. However, our data are the first to longitudinally describe an association between rapid declines in TNF-α concentrations and impaired clearance of a fungal infection in humans.

Our data have thereby extended the understanding of the mycological benefits of high baseline concentrations of proinflammatory cytokines, showing that even after establishment of effective antifungal therapy, persistently higher concentrations can be advantageous. In terms of clinical outcome, it was recently demonstrated that higher baseline concentrations of proinflammatory cytokines, including IFN-γ, are associated with reduced mortality in HIV-associated CM [22]. Our study supports those data, showing a similar effect on clinical outcomes: patients with good or intermediate outcomes by 10 weeks were more likely than those with poor outcomes, including death, to have a baseline IFN-γ concentration >30 pg/mL.

We found a limited effect of LTA4H genotype in CM. The TT LTA4H genotype has been associated with a hyperinflammatory phenotype, characterized by higher concentrations of TNF-α and IFN-γ, in adults with TBM in Vietnam [23]. We had only 20 patients with the TT LTA4H genotype in our study and thus lacked power to demonstrate an effect, although we found a similar trend. Lower baseline fungal counts have previously been linked to higher baseline concentrations of IFN-γ and TNF-α [22]; consistent with this finding, we found that patients with the TT genotype had lower fungal burdens. However, we did not identify a significant effect of the TT LTA4H genotype on mortality rate, or response to dexamethasone therapy, in HIV-associated CM.

The strengths of our study are that it was large, randomized, and longitudinal and included patients from both Africa and Asia, where the burden of CM is highest. This enabled a rigorous assessment of the effect of dexamethasone on immune responses. However, a significant proportion of patients had undetectable IFN-γ concentrations, which limited our analyses of this important biomarker. A more sensitive assay may have enabled more detailed analyses. Although our data suggested some heterogeneity in dexamethasone’s effect by LTA4H genotype, the study contained too few participants with the TT genotype to allow us to draw firm conclusions from this.

In conclusion, faster rates of decline in TNF-α concentration were associated with reduced fungal clearance, and dexamethasone led to faster rates of TNF-α decline (both absolute and relative to IL-10). This effect of dexamethasone therapy provides one explanation for the harmful effect of dexamethasone in HIV-associated CM. The body of evidence suggests that, in contrast to TBM and bacterial meningitis, proinflammatory immune responses are beneficial in HIV-associated CM. Our data support this conclusion and show that a proinflammatory response remains important for fungal clearance even once a patient is established on effective antifungal therapy.

## Supplementary Data

Supplementary materials are available at *Clinical Infectious Diseases* online. Consisting of data provided by the authors to benefit the reader, the posted materials are not copyedited and are the sole responsibility of the authors, so questions or comments should be addressed to the corresponding author.

Supplementary_Material

## Notes

***Acknowledgments.*** We thank trial participants, clinical, administrative, and laboratory staff at all sites.

***Disclaimer.*** The study funders had no role in study design, data collection, analysis, interpretation, or writing of the report.

***Financial support.*** This work was supported by the Wellcome Trust, the UK Department for International Development, the Medical Research Council (grant G1100684/1) Joint Global Health Trials program, part of the European and Developing Countries Clinical Trials Partnership, and a Wellcome Trust Intermediate Fellowship (grant WT097147MA to J. N. D).

***Potential conflicts of interest.*** Alll authors: No reported conflicts. All authors have submitted the ICMJE Form for Disclosure of Potential Conflicts of Interest. Conflicts that the editors consider relevant to the content of the manuscript have been disclosed.
