# Supplementary material for: Do Intracerebral Cytokine Responses Explain the Harmful Effects of Dexamethasone in Human Immunodeficiency Virus–associated Cryptococcal Meningitis?
Source: Clin Infect Dis. 2018 Aug 30;68(9):1494–501. doi: 10.1093/cid/ciy725 (PMC6481995; doi:10.1093/cid/ciy725)
Supplement: Supplementary_Material [file ciy725_suppl_supplementary_material.docx]

| **The two simple questions** | |
| --- | --- |
| Q1 | Does the patient require help from anybody for everyday activities? *(For example eating, drinking, washing, brushing teeth, going to the toilet.)* |
| Q2 | Has the illness left the patient with any other problems? |
| **The Modified Rankin Scale** | |
| **Grade** | **Description** |
| 0 | No symptoms |
| 1 | Minor symptoms not interfering with lifestyle |
| 2 | Symptoms that lead to some restriction in lifestyle, but do not interfere with the patients’ ability to look after themselves |
| 3 | Symptoms that restrict lifestyle and prevent totally independent living |
| 4 | Symptoms that clearly prevent independent living, although the patient does not need constant care and attention |
| 5 | Totally dependent, requiring constant help day and night |

**Supp Table 1** Disability grade according to two simple questions - Q1 answered “yes”: Poor outcome. Q2 answered “yes”: Intermediate outcome. If both questions answered “no”: Good outcome. Disability grade according to modified Rankin scale - Grade 0: Good outcome. Grade 1 or 2: Intermediate outcome. Grade 3-5: Poor outcome. The worst outcome from these two scores was used for analysis.

| **Characteristic** | **n** | **Cytokine study population** | **n** | **CryptoDex participants not included in the cytokine study** |
| --- | --- | --- | --- | --- |
|  |  | (N=274) |  | (N=176) |
| Gender | 274 |  | 176 |  |
| - Male sex |  | 173 (63%) |  | 106 (60%) |
| Age | 274 | 34 (30,40) | 176 | 37 (32,42) |
| Illness duration | 272 | 14 (7,28) | 187 | 12 (6,21) |
| GCS Category | 274 |  | 175 |  |
| - 10 or lower |  | 9 (3%) |  | 5 (3%) |
| - 11 to 14 |  | 47 (17%) |  | 25 (14%) |
| -15 |  | 218 (80%) |  | 145 (83%) |
| Opening Pressure (cmCSF) | 261 | 22 (14.9,32) | 142 | 24 (16,34) |
| CSF white cell count (cells / mm^3^) | 263 | 20 (5.01,44.7) | 162 | 26.91 (5.01,100) |
| CSF fungal count (log10) | 258 | 4.22 (2.18,5.49) | 168 | 4.38 (2.59,5.36) |

Supp Table 2 Baseline characteristics of cytokine study sub-population and residual CryptoDex population, displayed as median (IQR) for continuous data and n (%) for categorical data.

|  | **Clinical Outcome at 6 months** | | | |  |
| --- | --- | --- | --- | --- | --- |
| **Cytokine conc.** ***pg/***  ***ml*** | **Good**  **(N=57)** | **Intermediate (N=48)** | **Severe disability (N=14)** | **Death**  **(N=134)** | **p-value** |
| IFNγ |  |  |  |  | 0.05 |
| >30pg/ml | 23/56 (41%) | 12/48 (25%) | 3/14 (21%) | 28/131 (21%) |  |
| TNFα | 58.49 (30.91,111.43) | 48.17 (26.72,105.42) | 55.72 (10.78,222.86) | 41.36 (17.51,131.6) | 0.84 |
| MCP-1 | 1323.37 (776.05,3304) | 1209.34 (694.58,1884.54) | 1552.09 (989.12,3902.01) | 1663.49 (781.44,4067.71) | 0.17 |
| MIP-1a | 689.78 (461.44,1097.5) | 657.11 (410.15,1024) | 776.05 (404.5,1060.11) | 685.02 (404.5,1136.2) | 1.00 |
| GM-CSF | 4.56 (2,13.09) | 4.69 (2.16,11.24) | 4.03 (1.01,8.22) | 3.34 (1.09,10.93) | 0.48 |
| IL-6 | 294.07 (45.57,916.51) | 257.78 (35.75,1112.82) | 103.97 (33.59,867.07) | 118.6 (27.28,451.94) | 0.20 |
| IL-8 | 1332.57 (564.18,3821.7) | 1024 (491.14,3743.05) | 1937.53 (304.44,3691.52) | 1160.07 (380.04,2896.31) | 0.90 |
| IL-12 | 6.45 (2.93,11.96) | 7.52 (2.93,11.08) | 9.45 (2.93,12.21) | 7.06 (2.93,10.63) | 0.50 |
| IL-4 | 25.28 (16.34,31.12) | 25.81 (19.16,32.45) | 22.78 (19.43,33.13) | 23.92 (16.45,31.12) | 0.78 |
| IL-10 | 11.24 (3.94,26.35) | 10.41 (4.23,26.35) | 24.25 (2.68,41.64) | 9.19 (2.73,23.26) | 0.54 |
| IL-17 | 9 (4.92,17.03) | 8.06 (3.97,22.94) | 7.21 (1.82,11.96) | 6.5 (3.16,9.51) | 0.04 |

Supp Table 3 Comparison of baseline cytokine concentrations by clinical outcome at 6 months, shown as median (IQR) for continuous data and n (%) for categorical data. Statistical testing with the Wilcoxon rank-sum test for continuous data and Chi-square test for categorical data. IFN-γ concentrations dichotomised as above or below 30pg/ml and expressed as an odds ratio, because the majority of participants had concentrations below the lower limit of detection at baseline.

| **Relation between baseline features and 10 week mortality, based on logistic regression** | | | | |
| --- | --- | --- | --- | --- |
|  | OR | CI | Raw p-value | Adjusted p-value |
| Dexamethasone | 1.59 | (0.85-3.02) | 0.15 | 1.00 |
| IFNγ >30pg/ml | 0.38 | (0.15-0.91) | 0.03 | 0.50 |
| TNFα (log2 pg/ml) | 1.46 | (1.02-2.11) | 0.04 | 0.57 |
| MCP-1 (log2 pg/ml) | 1.31 | (1.03-1.69) | 0.03 | 0.45 |
| MIP-1a (log2 pg/ml) | 0.67 | (0.39-1.13) | 0.14 | 1.00 |
| GM-CSF (log2 pg/ml) | 1.01 | (0.78-1.29) | 0.96 | 1.00 |
| IL-6 (log2 pg/ml) | 0.90 | (0.75-1.09) | 0.27 | 1.00 |
| IL-8 (log2 pg/ml) | 1.16 | (0.86-1.59) | 0.34 | 1.00 |
| IL-12 (log2 pg/ml) | 0.92 | (0.63-1.31) | 0.63 | 1.00 |
| IL-4 (log2 pg/ml) | 1.13 | (0.77-1.69) | 0.54 | 1.00 |
| IL-10 (log2 pg/ml) | 1.03 | (0.80-1.33) | 0.79 | 1.00 |
| IL-17 (log2 pg/ml) | 0.73 | (0.57-0.93) | 0.01 | 0.19 |
| Baseline fungal count (log10 CFU/ml CSF) | 1.17 | (0.98-1.4) | 0.08 | 1.00 |
| Genotype TC vs TT | 0.58 | (0.08-3.94) | 0.57 | 1.00 |
| Genotype CC vs TT | 0.67 | (0.10-4.28) | 0.66 | 1.00 |

Table Supp4 Results of logistic regression on 10 week mortality by baseline cytokine concentrations, baseline fungal count, and LTA4H genotype

| **Cytokine slope** | **Correlation coefficient with early fungicidal activity**  **(95% CI)** |
| --- | --- |
| TNFα (log2 pg/ml/day) | -0.62 (-0.83 to -0.26) |
| IL-4 (log2 pg/ml/day) | -0.60 (-0.84 to -0.16) |
| IL-10 (log2 pg/ml/day) | -0.73 (-0.93 to -0.22) |

**Supp Table 5** Correlation coefficient (95% confidence interval) between cytokine slope and early fungicidal activity in the seven days following randomization. Negative values indicate an inverse relationship. Strong correlation > 0.7; moderate correlation 0.5-0.69; weak correlation 0.3-0.49.

|  | **TT Genotype** | **TC Genotype** | **CC Genotype** | **Comparison** |
| --- | --- | --- | --- | --- |
|  |  |  |  | (p-value) |
|  | N=7 | N=56 | N=156 |  |
| IFNγ (log2 OR) |  |  |  | 0.52 |
| - >30[pg/ml] | 3/7 (43%) | 17/56 (30%) | 40/152 (26%) |  |
| TNFα (log2 pg/ml) | 144.01 (45.25,200.85) | 48.84 (27.67,101.83) | 48.84 (19.56,131.6) | 0.55 |
| MCP-1 (log2 pg/ml) | 1845.76 (1060.11,4039.61) | 1595.73 (770.69,4299.64) | 1499.22 (776.05,3769.09) | 0.52 |
| MIP-1a (log2 pg/ml) | 942.27 (393.44,1144.1) | 749.61 (541.19,1144.1) | 689.78 (410.15,1060.11) | 0.64 |
| GM-CSF (log2 pg/ml) | 3.03 (0.75,23.59) | 3.48 (0.85,11.55) | 4.14 (1.48,11.47) | 0.77 |
| IL-6 (log2 pg/ml) | 141.04 (64.45,879.17) | 135.3 (30.48,448.82) | 171.25 (35.75,714.11) | 0.77 |
| IL-8 (log2 pg/ml) | 1629.26 (855.13,3983.99) | 1192.69 (471.14,3848.29) | 1160.07 (436.55,3236.01) | 0.82 |
| IL-12 (log2 pg/ml) | 6.32 (4.26,8.46) | 6.73 (2.93,9.71) | 7.16 (2.93,11.16) | 0.39 |
| IL-4 (log2 pg/ml) | 34.3 (13.64,35.02) | 28.44 (18.64,33.82) | 24.08 (16.22,30.7) | 0.31 |
| IL-10 (log2 pg/ml) | 8.28 (1.31,48.84) | 6.32 (1.87,17.75) | 10.85 (3.46,30.06) | 0.07 |
| IL-17 (log2 pg/ml) | 4.63 (4.2,7.94) | 6.96 (4.38,9.71) | 6.63 (3.16,11.63) | 0.85 |
|  | N=19 | N=112 | N=192 |  |
| Fungal count (log10 CFU/ml CSF) | 3.44(2.6,4.87) | 4.92(3.05,5.8) | 4.04(1.9,5.43) | 0.004 |
|  | N=20 | N=117 | N=194 |  |
| CSF white cell count (cells / mm^3^) | 19.3(1.99,55.7) | 17.99(5,68.72) | 20.1(5,45.15) | 0.46 |

**Supp Table 6** Comparison of baseline cytokine concentrations, white cell counts, and fungal counts by LTA4H genotype, shown as median (IQR) for continuous data and n (%) for categorical data. Statistical testing with the Wilcoxon rank-sum test for continuous data and Chi-square test for categorical data

|  | **TT Genotype** | **TC Genotype** | **CC Genotype** | **Comparison** |
| --- | --- | --- | --- | --- |
|  |  |  |  | (p-value) |
| Participants from Asia | N=17 | N=79 | N=64 |  |
| Fungal count (log10 CFU/ml CSF) | 3.44(2.64,4.75) | 5.08(3.53,5.92) | 5.10(3.10,5.79) | 0.03 |
|  |  |  |  |  |
| Participants from Africa | N=2 | N=33 | N=128 |  |
| Fungal count (log10 CFU/ml CSF) | 2.75(1.37,4.12) | 3.65(1.60,5.23) | 3.71(1.48,4.97) | 0.64 |

Supp Table 7 Fungal counts at baseline, by genotype, broken down by site. Statistical testing with the Wilcoxon rank-sum test.

| **Time-dependent hazard ratio for mortality**  **related to dexamethasone therapy, within each genotype** | | | | | |
| --- | --- | --- | --- | --- | --- |
|  | **Up to day 21** |  | **Day 22 - day 43** |  | **Day 43 - 180** |
|  | HR (CI) |  | HR (CI) |  | HR (CI) |
| **All** | 0.74 (0.48 to 1.12) |  | 2.80 (1.19 to 6.69) |  | 2.98 (1.49 to 5.97) |
| **CC** | 0.83 (0.49 to 1.43) |  | 3.20 (1 to 10.2) |  | 2.52 (1 to 6.3) |
| **CT** | 0.59 (0.28 to 1.21) |  | 2.67 (0.64 to 11.20) |  | 4.98 (1.52 to 16.32) |
| **TT** | 0.57 (0.09 to 3.535) |  | 1.4 (0.05 to 38.21) |  | 0.50 (0.02 to 10.16) |

Supp Table 8 Hazard ratios from Cox regression on 6 month mortality related to dexamethasone therapy, by genotype, with time-dependent variable to account for non-proportional hazards. Analysis corrected for participant’s country of origin.


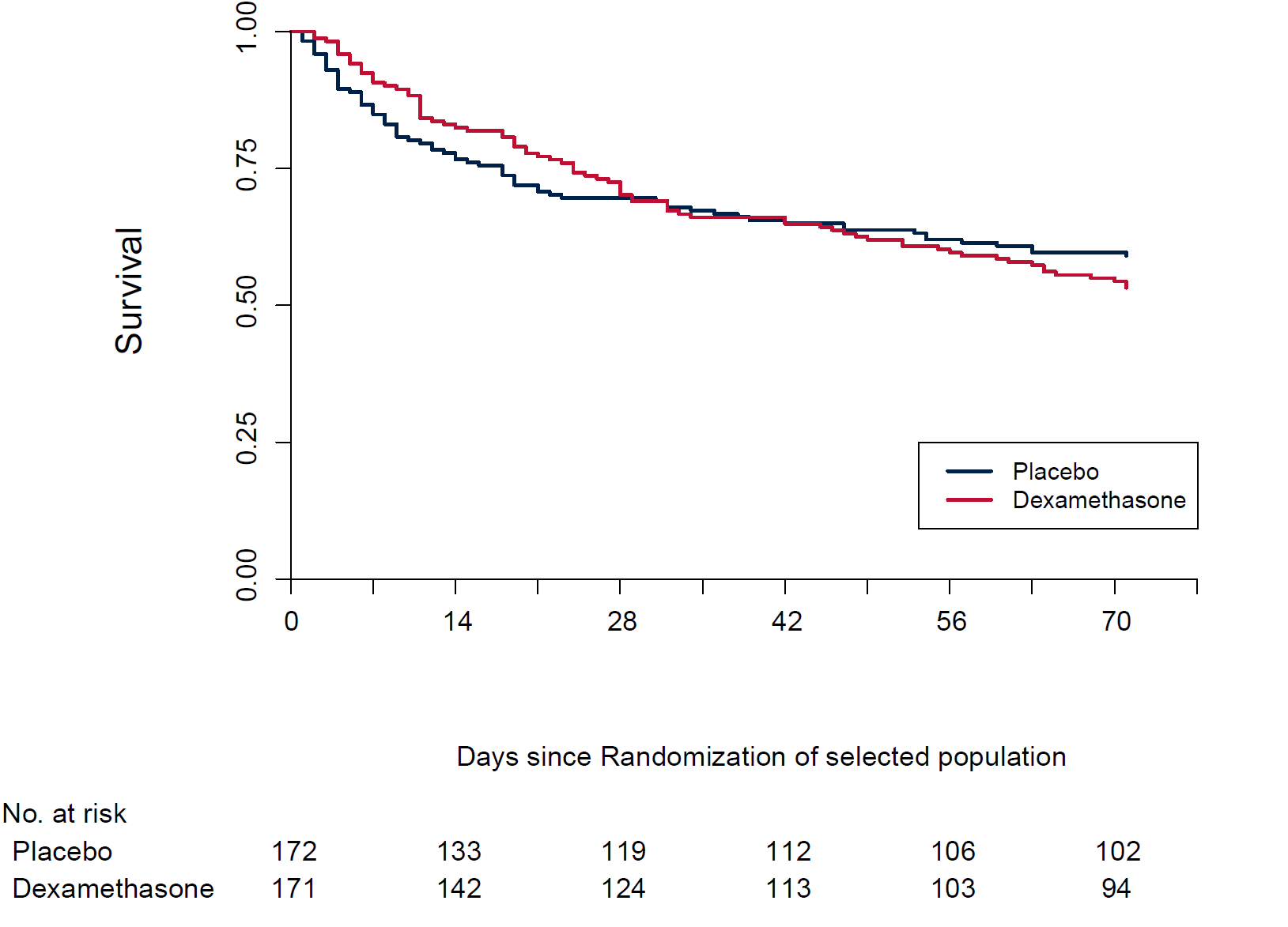


**Supp Fig 1a**


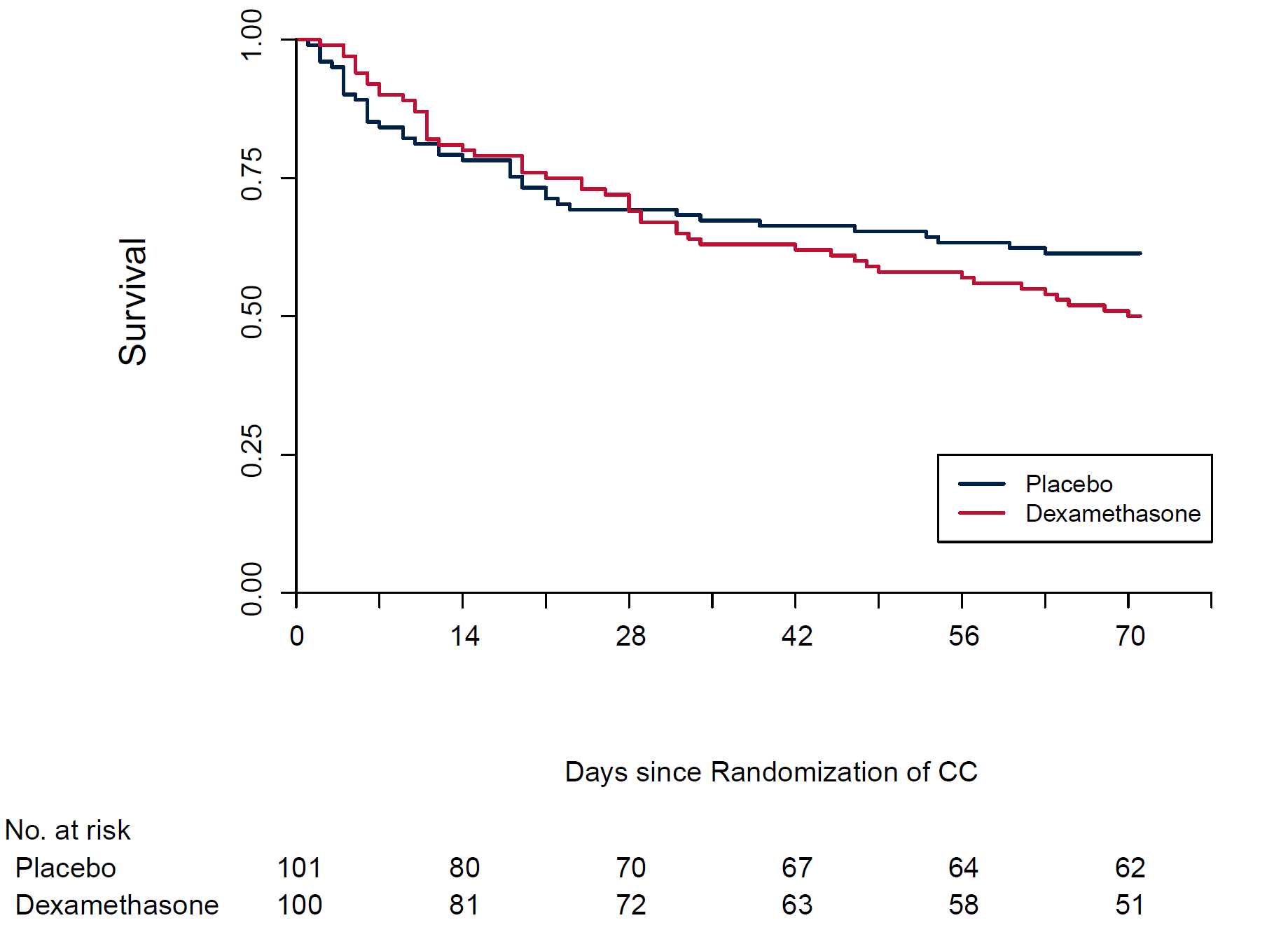


**Supp Fig 1b**


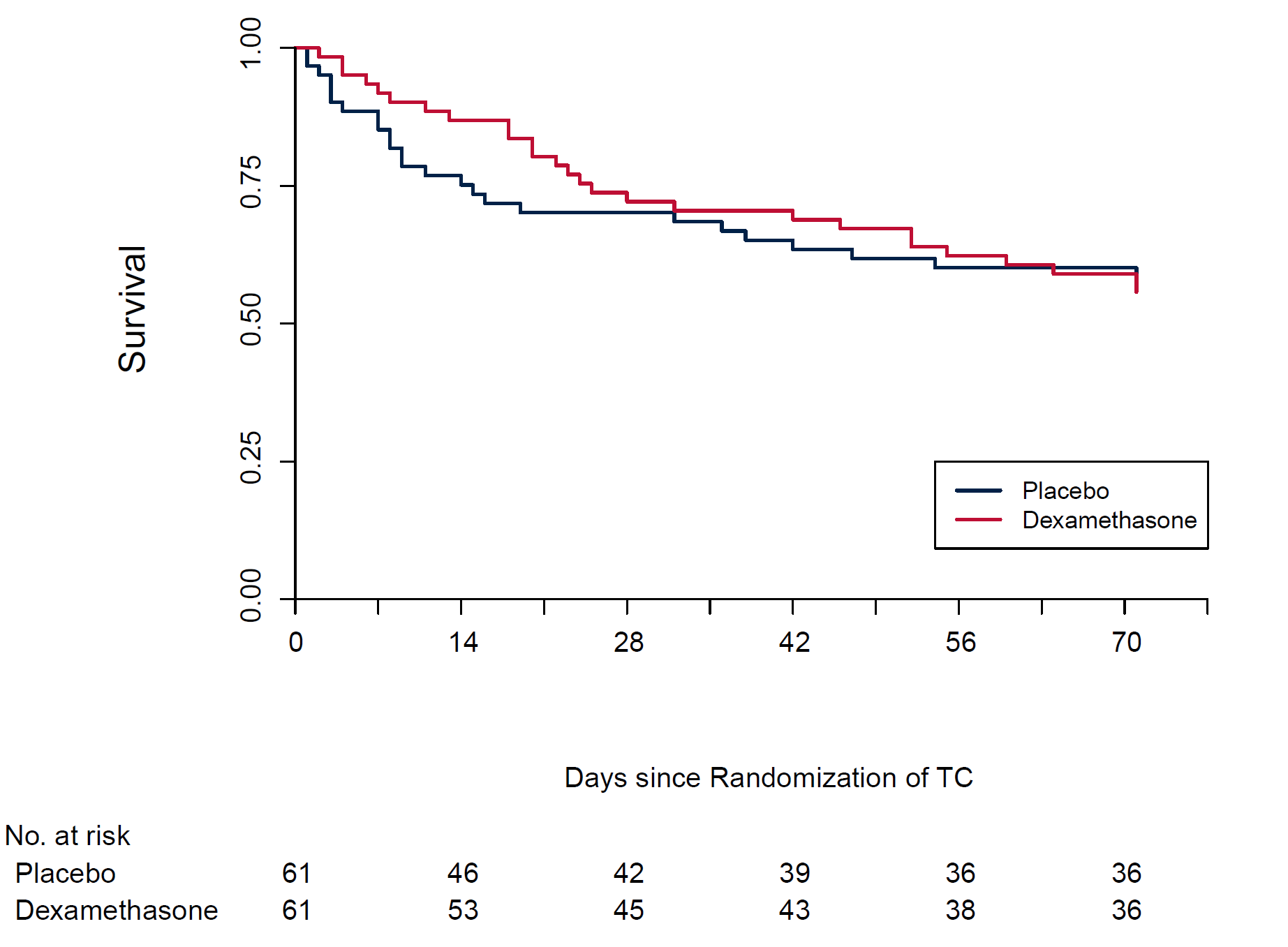


**Supp Fig 1c**


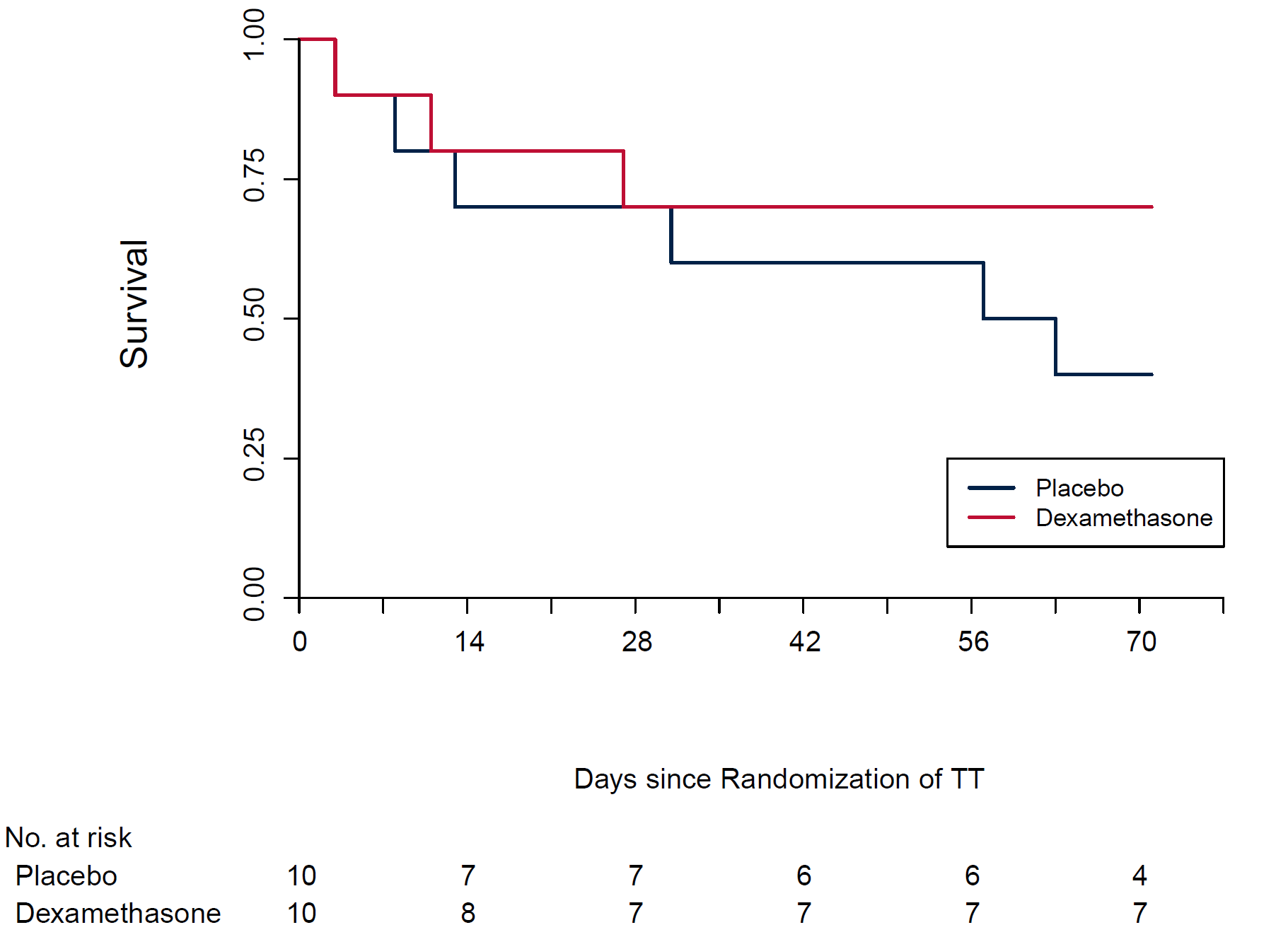


**Supp Fig 1d**

**Supp Fig 1a-d** Kaplan-Meier curves of survival up to 10 weeks in placebo (blue) and dexamethasone (red) arms. Displayed by all participants (a) and those with each of the three LTA4H genotypes: CC (b), CT (c), and TT (d).
